# The Statistical Foundations of Entropy

**DOI:** 10.3390/e23101367

**Published:** 2021-10-19

**Authors:** Petr Jizba, Jan Korbel

**Affiliations:** 1 Faculty of Nuclear Sciences and Physical Engineering, Czech Technical University, 11519 Prague, Czech Republic; petr.jizba@fjfi.cvut.cz; 2 Section for the Science of Complex Systems, Center for Medical Statistics, Informatics and Intelligent Systems (CeMSIIS), Medical University of Vienna, Spitalgasse 23, 1090 Vienna, Austria; 3 Complexity Science Hub Vienna, Josefstädterstrasse 39, 1080 Vienna, Austria

During the last few decades, the notion of entropy has become omnipresent in many scientific disciplines, ranging from traditional applications in statistical physics and chemistry, information theory, and statistical estimation to more recent applications in biology, astrophysics, geology, financial markets, or social networks. All these examples belong to the large family of complex dynamical systems that is typified by phase transitions, scaling behavior, multiscale and emerging phenomena, and many other non-trivial phenomena. Frequently, it turns out that in these systems, the usual Boltzmann–Gibbs–Shannon entropy and ensuing statistical physics are not adequate concepts. This especially happens in cases where the sample space in question does not grow exponentially.

This Special Issue, “The Statistical Foundations of Entropy”, is dedicated to discussing solutions and delving into concepts, methods, and algorithms for improving our understanding of the statistical foundations of entropy in complex systems, with a particular focus on the so-called generalized entropies that go beyond the usual Boltzmann–Gibbs–Shannon framework. The nine high-quality articles included in this Special Issue propose and discuss new tools and concepts derived from information theory, non-equilibrium statistical physics, and the theory of complex dynamical systems to investigate various non-conventional aspects of entropy with assorted applications. They illustrate the potential and pertinence of novel conceptual tools in statistical physics that, in turn, help us to *shed fresh light* on the statistical foundations of entropy.

In the first contribution [1], the authors discuss the important topic of ecological inference used to estimate individual behavior from the knowledge-aggregated data. The authors discuss two popular approaches: the Generalized Maximum Entropy approach and the Distributionally Weighted Regression equation. Moreover, the authors show that it is possible to obtain a combined solution with the so-called Generalized Cross-Entropy solution for the matrix adjustment problem.

The theory of critical phenomena is one of the essential parts of statistical physics with important overlaps to other scientific fields. The authors of the second paper [2] extend the classic Landau theory of critical phenomena in the context of multiscale dynamics. By using renormalization group theory, the authors can describe critical points through the inseparability of levels at certain points. These findings allow experimentalists more precise measurements of critical points.

The third contribution [3] describes a general approach toward generalized entropies based on the non-Newtonian calculus. The main idea is to redefine the usual arithmetic operations (e.g., addition and multiplication) and related calculus operators (e.g., differentiation and integration) in such a way that some crucial properties remain valid. Entropy and the corresponding thermodynamic quantities are then defined analogously to the Boltzmann–Gibbs–Shannon case in terms of these deformed operations. This approach incorporates many popular generalized entropies and gives a general recipe for the thermodynamics of such generalized entropies. Since the potential of this general approach is immense, the editors decided to promote this article by choosing the paper as the Editor’s Choice.

The aim of the fourth paper [4] was to show that for each distribution obtained from the principle of maximum entropy, there exists freedom in the choice of entropic functions and constraints, which make the reverse identification of entropic function and constraints from the form of MaxEnt distribution impossible. The paper consists of two simple examples of such invariance, where the different choices of entropy and constraints lead to the same MaxEnt distribution, which differs only in the Lagrange parameters. Since the Lagrange parameters may have some thermodynamic interpretation, it is essential to identify some additional properties of the system to decide which function plays the role of thermodynamic entropy.

In the fifth paper [5], the authors focused on the problem of estimation for entropy and the parameters of generalized Bilal distribution under an adaptive type II progressive hybrid censoring scheme. To this end, they used the maximum likelihood estimator and Newton–Raphson iteration method. In addition, some other estimators, such as the Bayesian estimator and confidence intervals, were also provided. Finally, the study contains an illustrative example that applies the obtained results.

The main result of the sixth contribution [6] was to introduce a generalization the concept of Shannon’s entropy power based on Rényi entropy. Consequently, the authors could generalize several popular identities, including the de Bruijn identity, isoperimetric inequality, or Stam inequality. Moreover, this enables one to introduce a new class of the one-parameter family of Rényi entropy power-based quantum mechanical uncertainty relations. These relations turn out to be very useful in many applications in quantum mechanics, including entanglement and quantum metrology.

The seventh paper [7] revisited the Boltzmann H theorem for both the classical and quantum systems. The authors considered a spatially inhomogeneous system initially out of equilibrium and studied its relaxation toward equilibrium. They accomplished this by considering small local cells and assuming the local equilibrium hypothesis. The global H-function is a sum of the local H functions. The authors recovered the H theorem for the case of spatially inhomogeneous gasses for both the classical and quantum cases. The correspondence principle connects the classical and quantum H functions.

In the eighth paper [8], the authors introduced a generalization of mutual information based on two-parameter Sharma–Mittal entropy. They also discussed the distinction between mutual information and capacity for the case of generalized entropies based on Sharma–Mittal entropy. They accomplished this by considering a proper axiomatic framework. Finally, the authors showed that the proper definition of both Sharma–Mittal mutual information and Sharma–Mittal capacity solves the issue of non-physical behavior.

Finally, in the last paper of this Special Issue [9], the authors reviewed the maximum entropy principle from the point of view of a general inference framework. The authors discussed a general procedure of updating the prior distribution to a posterior probability distribution through an eliminative induction process. The authors showed that under the assumption of subsystem independence, the logarithmic relative entropy (also called Kullback–Leibler divergence) is the unique solution that fulfills the prescribed axiomatic framework. Furthermore, the authors showed that this general framework contains the MaxEnt principle and Bayes’ rule as special cases and unifies the entropic and Bayesian inference methods.

Entropy is presumably one of the most intricate and complex scientific concepts. Its comprehension is an open challenge that requires new abstractions and methodological approaches. Information theory, statistical physics, and estimation theory methods provide a versatile and flexible framework with the potential to move research in this field forward. In this Special Issue, various conceptual and methodological approaches were provided to further deepen our understanding of the statistical foundations of entropy. They provided relevant pieces of research that addressed timely, current topics associated with the entropy paradigm. It is our hope that the reader will enjoy the articles included in this Special Issue and will find them helpful.
